# Transcription factor Ap2b regulates the mouse autosomal recessive polycystic kidney disease genes, *Pkhd1* and *Cys1*

**DOI:** 10.3389/fmolb.2022.946344

**Published:** 2023-01-12

**Authors:** Maoqing Wu, Naoe Harafuji, Amber K. O’Connor, Ljubica Caldovic, Lisa M. Guay-Woodford

**Affiliations:** ^1^ Center for Translational Research, Children’s National Hospital, Washington, DC, United States; ^2^ O’Neal Comprehensive Cancer Center, University of Alabama at Birmingham, Birmingham, AL, United States; ^3^ Center for Genetic Medicine Research, Children’s National Hospital, Washington, DC, United States; ^4^ Department of Genomics and Precision Medicine, School of Medical and Health Sciences, The George Washington University, Washington, DC, United States

**Keywords:** autosomal recessive polycystic kidney disease (ARPKD), *Cys1*, *Pkhd1*, *Tfap2b*, TFAP2B

## Abstract

Transcription factor Ap2b (TFAP2B), an AP-2 family transcription factor, binds to the palindromic consensus DNA sequence, 5′-GCCN_3-5_GGC-3’. Mice lacking functional *Tfap2b* gene die in the perinatal or neonatal period with cystic dilatation of the kidney distal tubules and collecting ducts, a phenotype resembling autosomal recessive polycystic kidney disease (ARPKD). Human ARPKD is caused by mutations in *PKHD1*, *DZIP1L*, and *CYS1,* which are conserved in mammals. In this study, we examined the potential role of TFAP2B as a common regulator of *Pkhd1* and *Cys1.* We determined the transcription start site (TSS) of *Cys1* using 5′ Rapid Amplification of cDNA Ends (5′RACE); the TSS of *Pkhd1* has been previously established. Bioinformatic approaches identified *cis*-regulatory elements, including two TFAP2B consensus binding sites, in the upstream regulatory regions of both *Pkhd1* and *Cys1*. Based on reporter gene assays performed in mouse renal collecting duct cells (mIMCD-3), TFAP2B activated the *Pkhd1* and *Cys1* promoters and electromobility shift assay (EMSA) confirmed TFAP2B binding to the *in silico* identified sites. These results suggest that *Tfap2b* participates in a renal epithelial cell gene regulatory network that includes *Pkhd1* and *Cys1*. Disruption of this network impairs renal tubular differentiation, causing ductal dilatation that is the hallmark of recessive PKD.

## 1 Introduction

Autosomal recessive polycystic kidney disease (ARPKD), characterized by bilateral cystic kidneys and congenital hepatic fibrosis, is a rare inherited disorder with a prevalence of 1:26,500 live births (Guay-Woodford et al., 2014; Alzarka et al., 2017). In the majority of patients, ARPKD is caused by mutations in the gene *PKHD1*, that encodes the protein fibrocystin (FPC) (Onuchic et al., 2002; Ward et al., 2002), the function of which is unknown. A major obstacle to understanding normal FPC function and the pathogenic mech**a**nisms that result from FPC deficiency is the lack of an orthologous animal model that fully recapitulates human *PKHD1*-associated renal disease (Outeda et al., 2017). *Pkhd1* deficient mice develop fibrocystic liver disease, but typically express either no renal phenotype or mild renal cystic disease (O’Connor and Guay-Woodford, 2015). By contrast, the congenital polycystic kidney (*cpk*) mouse strain, with a genetic defect in the *Cys1* gene, exhibits a phenotype that strikingly resembles human ARPKD (Hou et al., 2002; Nagao et al., 2012). *Cys1* encodes the protein cystin. Similar to other cystoproteins, both cystin and FPC localize to the primary apical cilium of renal epithelial cells (Tao et al., 2009). Recently, we described the first patient with ARPKD associated with *CYS1* mutations (Yang et al., 2021).

The AP2 family of transcription factors consists of five members designated TFAP2A, −B, −C, −D, and −E that influence promoter activity through binding to the consensus DNA sequence 5′-GCCN_3-5_GGC-3’ (Williams and Tjian, 1991a; Fornes et al., 2020). The AP2 family members are encoded by five genes that are found in both human and mouse genomes: *TFAP2A*/*Tfap2a*, *TFAP2B*/*Tfap2b*, *TFAP2C*/*Tfap2c*, *TFAP2D*/*Tfap2d*, and *TFAP2E*/*Tfap2e* (Hilger-Eversheim et al., 2000; Zhao et al., 2001; Cheng et al., 2002; Feng and Williams, 2003; Tummala et al., 2003; Zhao et al., 2003). All AP2 transcription factors share the same protein domain organization featuring a proline and glutamine rich transactivation domain at the N-terminus, followed by a basic DNA binding domain in the middle of the protein, and a helix-span-helix dimerization motif at the C-terminus (Williams and Tjian, 1991a; Williams and Tjian, 1991b; Moser et al., 1995; Eckert et al., 2005). AP2 family members can form both homo- and heterodimers that regulate gene expression (Eckert et al., 2005). All AP2 genes are expressed early in the developing mouse central nervous system, but later in embryonic development, each family member exhibits a tissue-specific expression pattern (Moser et al., 1997b; Eckert et al., 2005).

TFAP2B is expressed in developing and adult kidneys (Moser et al., 1995; Moser et al., 1997b). Single cell RNA-Seq analysis using mouse kidneys revealed that *Tfap2b* is expressed primarily in the cortical collecting duct cells (Park et al., 2018; Lamontagne et al., 2022). TFAP2B expression is associated with distal tubule cell fate determination (Miao et al., 2021). In humans, germline mutations of *TFAP2B* cause Char syndrome, an autosomal dominant disorder characterized by patent ductus arteriosis, digital abnormalities and facial dysmorphism (Raap et al., 2021). While TFAP2B can be detected in distal tubules of the adult kidney, there is no human renal phenotype, suggesting involvement of additional factors in human renal tubular differentiation. By contrast, in mice, *Tfap2b* gene disruption leads to a renal phenotype with cystic dilatation of distal tubules and collecting ducts, in a pattern that phenocopies recessive PKD (Moser et al., 1997a; Moser et al., 2003; Wang et al., 2018; Lamontagne et al., 2022). The observations linking *Tfap2b* with renal cystogenesis, together with its expression during kidney development, led us to speculate that TFAP2B may regulate the expression of mouse cystogenes, particularly those implicated in ARPKD. The paradigm of renal cystic disease linked to a transcription factor mutation with altered expression of multiple cystogenic genes has been previously established. Mutations in the gene encoding hepatocyte nuclear factor-1-beta (HNF1B) cause cystic kidney disease, and HNF1B has been shown to regulate expression of several cystogenic genes, including *Pkhd1* and *Cys1* (Shao et al., 2020).

In the current study, we evaluated whether TFAP2B regulates the expression of ARPKD-associated genes *Pkhd1* and *Cys1* in the inner medullary collecting duct cell line, mIMCD-3, a widely used experimental cell model. We identified putative TFAP2B binding sites in the upstream regulatory regions of both *Pkhd1* and *Cys1* using bioinformatic approaches. Binding sites identified *in silico* were then tested experimentally using reporter gene and electrophoretic mobility shift assays (EMSA) to show that *Pkhd1* and *Cys1* are indeed regulated by TFAP2B.

## 2 Materials and methods

### 2.1 Bioinformatic analysis of mouse *Cys1* and *Pkhd1* promoters

Grep command and regular expressions that describe TFAP2B consensus binding sites (Supplementary Figure S1) in the JASPAR database of transcription factor binding sites (Fornes et al., 2020) were used to query both sense and antisense strands of the *Cys1* and *Pkhd1* upstream regulatory regions, chr12:24681631-24684806 and chr1:20618038-20620038 of the GRCm38/mm10 mouse genome assembly, respectively.

### 2.2 RT-PCR of *Tfab2b* mRNA isoforms from kidneys and mIMCD-3 cells

Kidney tissue from a 6-week-old female mouse was snap frozen, transferred to a gentleMACS M tube (Miltenyi Biotec, Cat. No. 130-093-236, RRID:*SCR_020269*) in Buffer RLT plus 2-Mercaptoethanol (as per RNeasy Mini Kit instructions, see below) and homogenized using a gentleMACS Dissociator (Miltenyi Biotec) using manufacturer’s program RNA-02. Homogenized samples were transferred to microcentrifuge tubes for total RNA extraction using the RNeasy Mini Kit (Qiagen, Cat. No. 74104) according to manufacturer’s instructions.

mIMCD-3 cells were purchased from American Type Culture Collection (ATCC, Cat. No. CRL-2123, RRID:*CVCL_0429*), and maintained in DMEM/F-12 medium (Thermo Fisher Scientific, Cat. No. 11330057) containing 10% heat-inactivated fetal bovine serum (Atlanta Biologicals, Cat. No. S11050H) and 1% penicillin/streptomycin (Thermo Fisher Scientific, Cat. No. 15140163). Cells were cultured at 37°C in 5% CO_2_/95% air. Total RNA was isolated from cells using the RNeasy Mini Kit (Qiagen) according to the manufacturer’s instructions.

RNA was treated with RQ1 RNase-Free DNase (Promega, Cat. No. M6101), and then repurified using the RNeasy Mini kit. For RT-PCR, RNA samples were reverse-transcribed using SuperScript III First-Strand Synthesis SuperMix (Thermo Fisher Scientific, Cat. No. 18080400) and oligo dT primers. PCR was performed using primer sets specific for each *Tfap2b* isoform (Supplementary Table S1), GoTaq Master Mix (Promega, Cat. No. M7123), .2 µM forward and reverse primer, and either 4 ng of cDNA in the first round of PCR or .4 µL of amplification product in nested PCR using the PCR conditions listed in Supplementary Table S2.

### 2.3 Plasmid construction

DNA fragments harboring presumptive upstream regulatory regions of the mouse *Cys1* gene (Table 2) were generated by PCR using 300 nM primers listed in Supplementary Table S3, Expand Long Template Enzyme mix (Roche Cat. No. 1173264100), 350 µM dNTPs, 200 ng of genomic DNA from C57BL/6j mice as template, and amplification conditions listed in Supplementary Table S4. Amplification products were subcloned into the pGL3-basic reporter gene plasmid (Promega) using *Nhe*I and *Hind*III restriction enzymes for *Cys1*p-constructs. DNA fragments harboring presumptive upstream regulatory regions of the mouse *Pkhd1* gene (Table 2) were generated by PCR using 300 nM primers listed in Supplementary Table S5, 1x Expand Long Template Enzyme mix, 350 µM dNTPs, 200 ng of genomic DNA from C57BL/6j mice as template, and amplification conditions listed in Supplementary Table S4. Amplification products for constructs *Pkhd1*p-1443 and *Pkhd1*p-1434 were cloned into pGL3-basic reporter gene plasmid (Promega) using restriction enzymes *Acc*65I and *Bgl*II, while *Bgl*II and *Hind*III were used for constructs *Pkhd1*p-981, *Pkhd1*p-950 and *Pkhd1*p-941.

To generate *Tfap2b1* and *Tfap2b2* expression vectors, the RNeasy Mini Kit (Qiagen) was used to isolate total RNA from 5 days post-confluent mIMCD-3 cells cultured as described above. Superscript First-strand Synthesis (Thermo Fisher Scientific, Cat. No. 11904-018) was used for cDNA generation following manufacturer’s instructions. Full-length *Tfap2b1* and *Tfap2b2* coding sequences were amplified from mIMCD-3 cDNA using primers 5′-GGG GTA CCC ACT CAC CTC CTA GAG ACC AGG-3’ and 5′-CCT TAA TTA AGC TCA TTT CCT GTG TTT CTC CTC CTT GTC-3′, and 5′-CGG AAT TCA CCA TGG CAT TAG TCC ACA CCT ATT CAT CCA TGG-3’ and 5′-CCT TAA TTA AGC TCA TTT CCT GTG TTT CTC CTC CTT GTC-3′, respectively, and Expand High Fidelity PCR System (Roche Cat. No. 11732641001), 200 µM each dNTP, 500 ng of cDNA, and the following amplification conditions: 2 min initial denaturation at 94°C, 35 cycles of 10 s denaturation at 94°C, 30 s annealing at 62°C, 50 s elongation at 72°C, and 5 min final elongation at 72°C. The *Tfap2b1*, *Tfap2b2* amplification products were subcloned into pCMV-Myc-N (Clontech Laboratories Inc. Cat. No. 635689) expression plasmid using *EcoR*I and *Not*I restriction enzymes to yield the expression plasmids CMV-Tfap2b1 and CMV-Tfap2b2.

### 2.4 5′ Rapid amplification of cDNA ends (5′ RACE)

5′ RACE was performed using FirstChoice RLM-RACE (Ambion, Cat. No. 1700). Total RNA was extracted from 5 days post-confluent mIMCD-3 cells using RNeasy Mini Kit (Qiagen) according to the manufacturer’s instructions (and see above). RNA was treated with Calf Intestine Alkaline Phosphatase followed by Tobacco Acid Pyrophosphatase. A 45 nt RNA adapter oligonucleotide 5-GCU GAU GGC GAU GAA UGA ACA CUG CGU UUG CUG GCU UUG AUG AAA-3′ was ligated to the decapped mRNA using T4 RNA ligase, followed by reverse transcription using random hexamers. cDNA was generated and used as a template for nested PCR using first, the 5′ RACE outer adaptor primer 5′-GCT GAT GGC GAT GAA TGA ACA CTG-3′ and 3′ *Cys1*-specific outer primer 5′-GGG TGG GAG TCA TGC TGG GAG CAA G-3′, followed by PCR with the inner 5′ RACE adaptor primer 5′-CGC GGA TCC GAA CAC TGC GTT TGC TGG CTT TGA TG-3′ and 3′ *Cys1*-specific inner primer 5′-CCC AAG CTT GGA GAC TAG CAC TGT CGG AAA GGA GG-3′ to amplify the 5′ products containing potential *Cys1* transcription start sites. PCR products were separated by agarose gel electrophoresis, isolated and purified from the gel, cloned into the pCR2.1-TOPO plasmid using the TOPO TA Cloning Kit, and used to transform One Shot TOP10 chemically competent *E. coli* cells (Thermo Fisher Scientific, Cat. No. K450001). Individual PCR products from single clones were then sequenced and mapped onto the mouse genomic DNA sequence.

### 2.5 Site-directed mutagenesis

QuikChange Site-Directed Mutagenesis (Stratagene, Cat. No. 200522) was used to generate altered promoter constructs. Design and use of mutagenic oligonucleotide primers was per manufacturer’s specifications and instructions. Mutations were confirmed in all constructs by sequence analysis.

### 2.6 Reporter gene assays

mIMCD-3 cells (ATCC) grown to 90%–95% confluence in 24-well plates were transfected using Lipofectamine 2000 Transfection Reagent (Thermo Fisher Scientific, Cat. No. 11668030). The transfection efficiency was normalized using the *Renilla* pRL plasmid (15 ng), and the total quantity of transfected DNA was kept constant using pcDNA3.1 (+) Mammalian Expression Vector (Thermo Fisher Scientific, Cat. No. V79020). Between 48 and 72 h after transfection, luciferase activity was measured using the Dual-Luciferase Reporter Assay System (Promega, Cat. No. E1910) according to the manufacturer’s instructions. The luminometer was programmed to perform a 2-s premeasurement delay, followed by a 10-s measurement period for each reporter assay. After measurement of the firefly luciferase activity, Stop & Glo Reagent was added to each sample and enzymatic activity of *Renilla* luciferase was measured.

### 2.7 Electrophoretic mobility shift assay (EMSA)

Electrophoretic mobility shift assay (EMSA) was performed as previously described (Wu et al., 2013). Briefly, nuclear extracts, predicted to contain endogenous TFAP2B, were prepared from 5-day post-confluent mIMCD-3 cells using Nuclear and Cytoplasmic Extraction kits (Pierce, Cat. No. 78833). Biotin-labeled oligonucleotides were prepared as we described (Wu et al., 2013). Binding reactions were performed according to the instructions provided with the LightShift Chemiluminescent EMSA kit (Pierce, Cat. No. 20148). Samples were run on non-denaturing polyacrylamide gels. Biotin-labeled DNA oligonucleotides were detected with avidin-based Chemiluminescent Nucleic Acid Detection kit (Pierce, Cat. No.89880). Reduction in the mobility of protein-oligonucleotide complexes on the gels represented the binding of TFAP2B to the oligonucleotide.

## 3 Results

### 3.1 Identification of the *Cys1* transcription start site (TSS)

**FIGURE 1:**
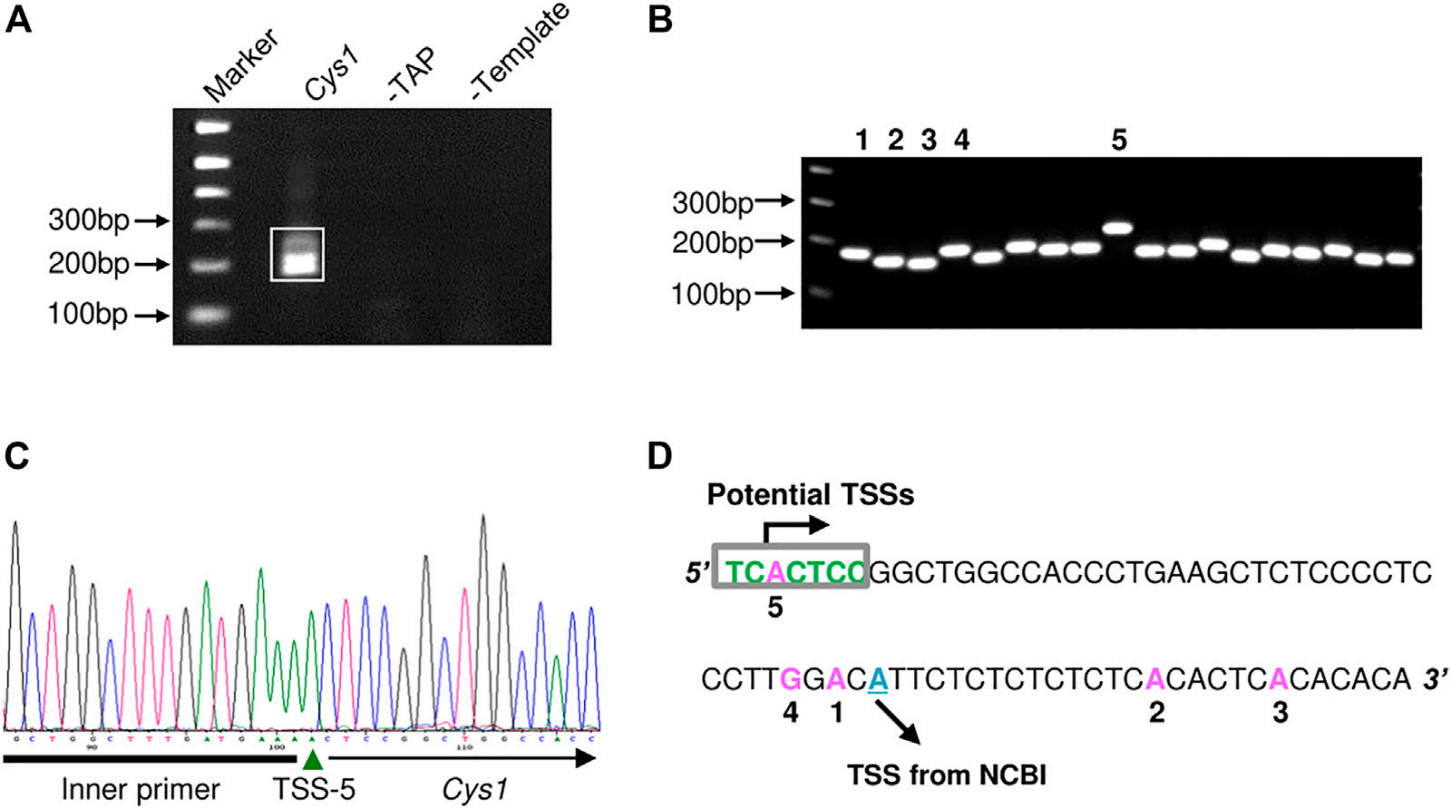
Determination of the *Cys1* transcription start site (TSS) using 5′ RACE. **(A)** Agarose gel electrophoresis of *Cys1* 5′ RACE PCR products. The boxed PCR reaction products (lane *Cys1*) were cloned and sequenced. Remaining lanes show the results of negative control PCR reactions: middle lane, template mRNA was not decapped by TAP treatment (-TAP, a negative control for RACE); right lane, non-reverse transcribed mRNA subjected directly to PCR (-Template, a negative control for PCR amplification). **(B)** Individual colony-based PCR reaction products generated using cloned *Cys1* amplicon plasmid DNA as template. Clones 1–5 were analyzed by DNA sequencing. **(C)** Clone 5 DNA sequencing data. Inner primer indicates sequence corresponding to the 5′ RACE adaptor primer from the FirstChoice RLM-RACE kit. **(D)** Locations of TSS from the five clones are shown in pink; initiator consensus sequence [PyPyAN (T/A)PyPy] is shown in green (Py denotes pyrimidine, C or T). Only the candidate TSS from clone 5 fully conforms to the initiator consensus sequence. The putative TSS from NCBI is depicted in cyan.

The proximal regulatory regions of *Pkhd1* and *Cys1* extend upstream from the transcription start sites (TSS). The TSS of *Pkhd1* has been identified (Hiesberger et al., 2004), therefore we used 5′ rapid amplification of cDNA ends (5′ RACE) to identify the *Cys1* TSS. A *Cys1* gene-specific reverse primer annealing within the first exon and an adaptor forward primer were used to amplify the 5′ ends of *Cys1* cDNAs prepared from mouse inner medullary collecting duct cell line (mIMCD-3) total RNA (Figure 1A). We cloned and sequenced five distinctly-sized 5′ RACE products (Figures 1B–D). Only the 5′ RACE product representing the longest transcript fully conforms to the consensus TSS (PyPyA (T/A)PyPy) (Haberle and Stark, 2018). The four other products failed to match the consensus sequence, and that was also true of a TSS identified in NCBI (Figure 1D). Based on our findings, *Cys1* may have more than one TSS, and we note that DNA upstream of *Cys1* includes a GC-rich region, a feature common among genes with multiple TSSs (Shao et al., 2020). Throughout we refer to the TSS defined by the longest transcript in assigning *Cys1* DNA sequence position designations.

### 3.2 Identification of TFAP2B binding sites in mouse *Cys1* and *Pkhd1* upstream regulatory regions

**FIGURE 2:**
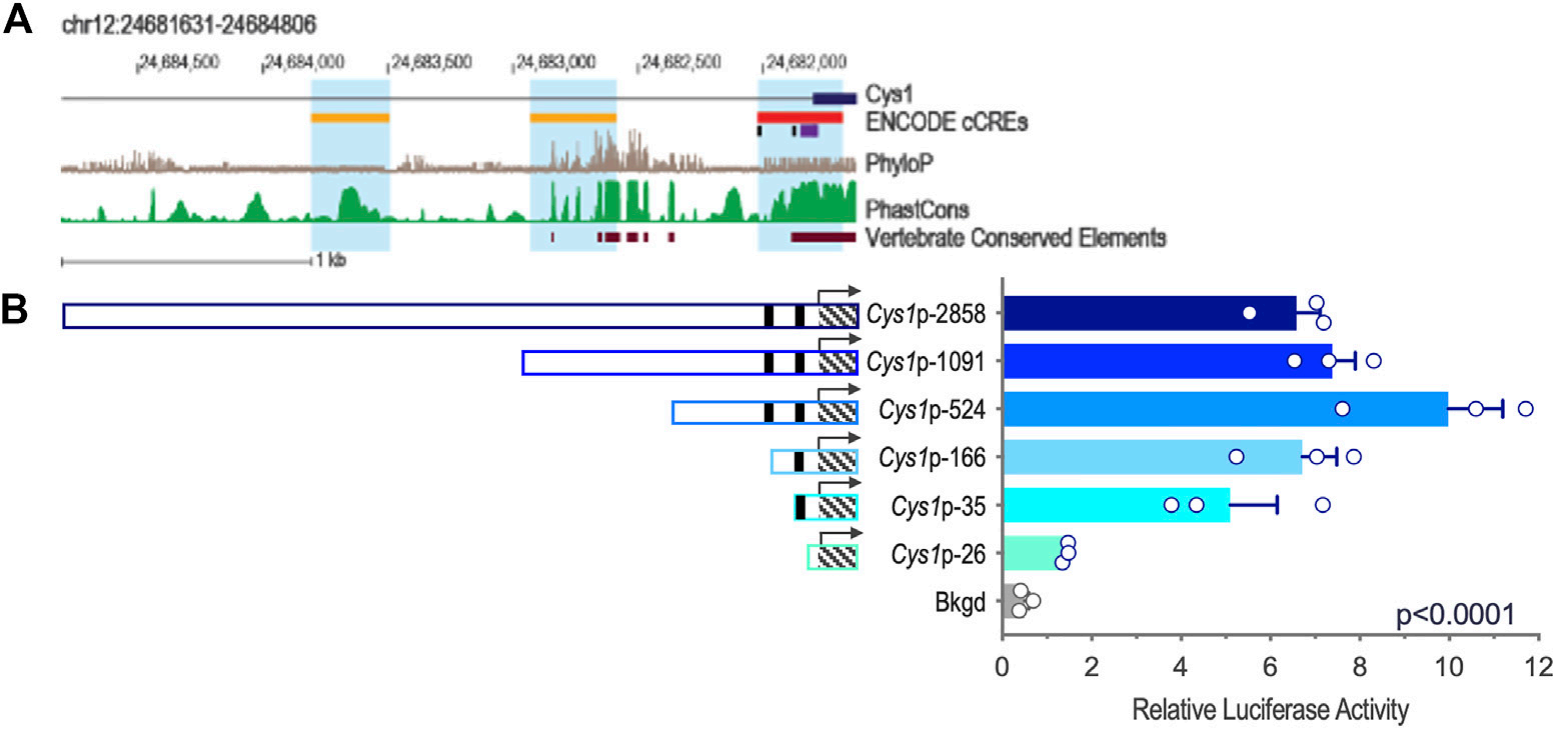
*Cys1* upstream regulatory region and reporter gene analysis of *Cys1* promoter constructs. **(A)** Map of the *Cys1* upstream regulatory region showing UCSC tracks for predicted regulatory elements (cCRE and Vertebrate Conserved Elements) and sequence conservation (phyloP and PhastCOns). Black rectangles indicate TFAP2B binding sites. **(B)** *Cys1* promoter constructs and corresponding relative luciferase activities in transfected mIMCD-3 cells. Black rectangles indicate TFAP2B binding sites. Hatched boxes indicate *Cys1* 5′-UTR sequences. Arrows indicate TSS. The *Cys1* TSS genomic coordinate is Chr12:24,681,806 from the GRCm38/mm10 mouse genome assembly. The error bars indicate S.E.M. One-way ANOVA was used to determine the significance of differences among mean luciferase activities associated with each construct.

**FIGURE 3:**
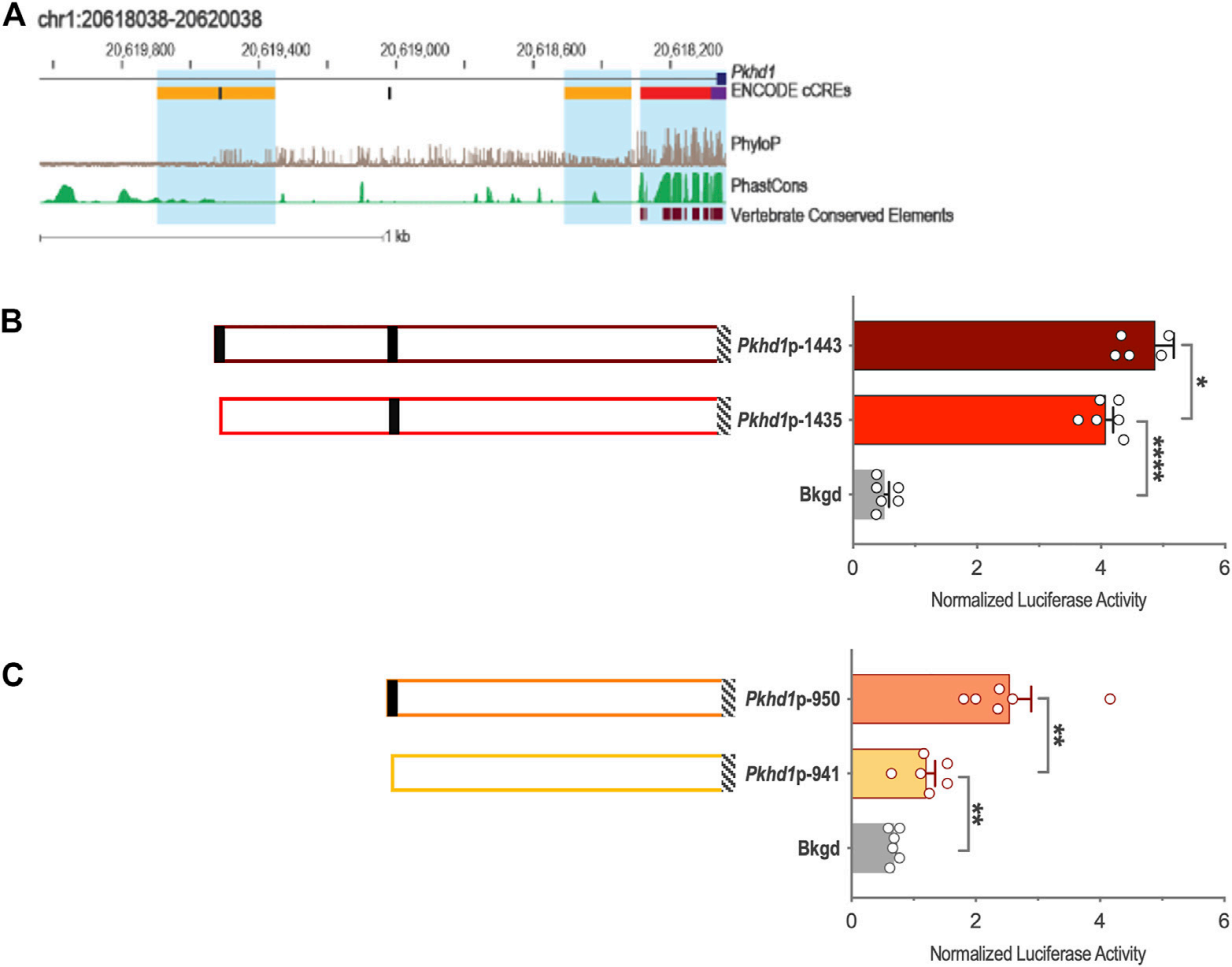
*Pkhd1* upstream regulatory region and reporter gene analysis of the *Pkhd1* promoter constructs. **(A)** Map of the *Pkhd1* upstream regulatory region showing UCSC tracks for predicted regulatory elements (cCRE and Vertebrate Conserved Elements) and sequence conservation (phyloP and PhastCOns). Black rectangles indicate TFAP2B binding sites. **(B and C)** *Pkhd1* promoter constructs and corresponding normalized luciferase activities in transfected mIMCD-3 cells. Black rectangles indicate TFAP2B binding sites. Hatched boxes indicate *Pkhd1* promoter and 5′-UTR sequences. The error bar indicates S.E.M.; * indicates *p* < .05; ** indicates *p* < .01.

Genomic DNA sequences spanning 3,175 and 2,000 bp upstream of the *Cys1* and *Pkhd1* TSSs, respectively, were analyzed using bioinformatics approaches to identify candidate TFAP2B binding sites (Supplementary Figure S1) (Fornes et al., 2020). Both *Cys1* and *Pkhd1* regulatory regions were each found to harbor two TFAP2B binding sites, at −175 and −35 for *Cys1*, and −1443 and −950 for *Pkhd1* (Table 1). Inspection of the UCSC Genome Browser conservation tracks (Felsenstein and Churchill, 1996; Siepel et al., 2005; Pollard et al., 2010), ENCODE candidate *cis*-regulatory elements (cCRE) (Leung et al., 2015) and vertebrate conserved elements (Chiaromonte et al., 2002) revealed predicted promoter regions and regulatory elements upstream of *Cys1* and *Pkhd1* TSSs (Figure 2A; Figure 3A). Three of the four candidate TFAP2B binding sites are located within the predicted regulatory elements of *Cys1* and *Pkhd1* (Figure 2A, Figure 3A). For *Cys1*, both TFAP2B binding sites are located within the predicted promoter region (Figure 2A, black rectangles). In the case of *Pkhd1*, one TFAP2B binding site is located within a predicted enhancer, while the second is between predicted enhancers (Figure 3A, black rectangles). These data are consistent with the reported renal cystic phenotype of mice deficient in TFAP2B (Moser et al., 1997a; Moser et al., 2003; Wang et al., 2018).

**TABLE 1:** Predicted TFAP2B binding sites in the *Cys1* and *Pkhd1* promoters.

Gene	Position of TFAP2B binding sites	Sequence	Strand
*Cys1*p	Proximal: −35 to −26	GCCCAGGGC	sense
	Distal: −175 to −166	GCCGGGGGG	anti-sense
*Pkhd1*p	Proximal: −950 to −942	GCCCCAGGC	sense
	Distal: −1443 to −1435	GCCCTTGGC	sense
consensus	—	GCCNNNGGC	sense

### 3.3 Expression of *Tfap2b* derived mRNAs in mouse kidney and collecting duct cells

Query of the NCBI database revealed that mouse *Tfap2b* mRNA is alternatively spliced to produce three transcripts corresponding to three protein isoforms. The *Tfap2b* transcripts NM_009334.3 and NM_001025305.2 that encode TFAP2B1 (NP_033360.2) and TFAP2B2 (NP_001020476.1) isoforms are 6132 and 6167 nt in length, respectively, while the TFAP2B3 (NP_001273269.1) isoform is encoded by 3813 nt transcript NM_001286340.1. We focused our studies on the TFAP2B1 and TFAP2B2 isoforms because their transcripts correspond in size to *Tfap2b* mRNA reported in developing mice (Moser et al., 1995). We used isoform-specific RT-PCR to confirm the expression of Tfap2b derived transcripts in mouse kidney and in the mIMCD-3 cell line (Supplementary Figure S2). Single round of RT-PCR amplification using kidney tissue was sufficient to detect *Tfap2b1* and *Tfap2b2* transcripts while nested PCR was required to detect *Tfap2b3* transcript (Supplementary Figure S2). In mIMCD-3 cells, *Tfap2b1* and *Tfap2b2* transcripts were detected using nested PCR, but no *Tfap2b3* transcript was detectable (Supplementary Figure S2). Although we did not perform a quantitative RT-PCR analysis, our results suggest that expression levels of *Tfap2b1* and *Tfap2b2* isoforms exceed levels of *Tfap2b3* in mouse kidneys, and that in mIMCD-3 cells, only *Tfap2b1* and *Tfap2b2* isoforms are expressed at levels detectable by PCR.

### 3.4 Deletion analysis of *Cys1* and *Pkhd1* upstream regulatory regions

We tested whether the predicted *Cys1* and *Pkhd1* upstream regulatory elements, including TFAP2B binding sites, influenced gene expression. Deletion fragments of *Cys1* and *Pkhd1* upstream DNA (Table 2) were linked to a luciferase reporter gene and assayed in mIMCD-3 cells expressing *Tfap2b1* and *Tfap2b2* mRNAs. Six *Cys1* promoter reporter constructs were tested, spanning genomic regions −2858, −1091, −524, −166, −35, and −26 to +175 bp (Table 2; Figure 2B). Reporter activity directed by *Cys1*p-35 with one TFAP2B binding site was notably increased relative to the *Cys1*p-26 construct lacking a TFAP2B binding site (Figure 2B), suggesting that TFAP2B indeed regulates *Cys1* gene expression by binding to its promoter. Further evidence of a potential role for TFAP2B in *Cys1* regulation was demonstrated by a notable difference in luciferase activities between *Cys1*p-166, which harbors one TFAP2B binding site, and *Cys1*p-524 with two TFAP2B binding sites (Figure 2B), suggesting that both TFAP2B binding sites play roles in activating *Cys1* gene expression. Luciferase activities derived from *Cys1*p-524 were the highest, while luciferase activities derived from *Cys1*p-1091 and *Cys1*p-2858 constructs were lower (Figure 2B). These findings suggest the presence of negative regulatory elements between *Cys1*p-524 and *Cys1*p-2858 of the *Cys1* upstream regulatory region.

**TABLE 2:** *Cys1* and *Pkhd1* genomic DNA regions cloned into pGL3.

Plasmid name	Genomic region (GRCm38/mm10 mouse genome assembly)	Coordinates relative to TSS
*Cys1*p-2858	Chr12:24,681,620-24,684,653	−2858 to +175
*Cys1*p-1091	Chr12:24,681,620-24,682,886	−1091 to +175
*Cys1*p-524	Chr12:24,681,620-24,682,319	−524 to +175
*Cys1*p-166	Chr12:24,681,620-24,681,961	−166 to +175
*Cys1*p-35	Chr12:24,681,620-24,681,830	−35 to +175
*Cys1*p-26	Chr12:24,681,620-24,684,821	−26 to +175
*Pkhd1*p-1443	Chr1:20,618,039-20,619,498	−1443 to +19
*Pkhd1*p-1434	Chr1:20,618,039-20,619,489	−1434 to +19
*Pkhd1*p-981	Chr1:20,618,039-20,619,036	−981 to +19
*Pkhd1*p-950	Chr1:20,618,039-20,619,005	−950 to +19
*Pkhd1*p-941	Chr1:20,618,039-20,618,996	−941 to +19

To functionally assess TFAP2B regulation of *Pkhd1* gene expression, we generated four reporter gene constructs containing genomic DNA regions −1443, −1435, −950, and −941 to +19 (Table 2; Figures 3B, C) relative to the *Pkhd1* TSS. Using reporter gene assays as described above, we compared luciferase activity in mIMCD-3 cells after transfection of constructs lacking one or both TFAP2B binding sites (Figures 3B, C). Removal of the distal TFAP2B binding site resulted in lower luciferase activity, as indicated by comparison of *Pkhd1*p-1443 vs. *Pkhd1*p-1435 transfected cells (Figure 3B). Similarly, removal of the proximal TFAP2B binding site led to reduced luciferase activity as shown by comparison of *Pkhd1*p-950 to *Pkhd1*p-941 transfected cells (Figure 3C). These data are consistent with the presence of functional TFAP2B binding sites within the *Pkhd1* upstream regulatory region and that TFAP2B can regulate *Pkhd1* transcription.

Taken together, our results support the role of TFAP2B in the activation of both *Pkhd1* and *Cys1* transcription. These findings in turn support the hypothesis that *Pkhd1* and *Cys1* expression are downregulated in *Tfap2b* ^−/−^ cystic mice, in a manner analogous to HNF1B deficiency leading to reduced cystogenic gene expression and a cystic phenotype (Shao et al., 2020). Further studies are required to test this hypothesis *in vivo*, including a careful analysis of the potential for *Cys1* transgene overexpression to confer partial or complete rescue of the renal cystic phenotype in *Tfap2b* ^*−/−*^ mice, as was previously demonstrated for *Cys1* transgene rescue of renal cystic disease in *Cys1* ^*cpk/cpk*^ (*cpk*) mice (Yang et al., 2021).

### 3.5 *Cys1* and *Pkhd1* promoters are both regulated by TFAP2B1 and TFAP2B2 isoforms

**FIGURE 4:**
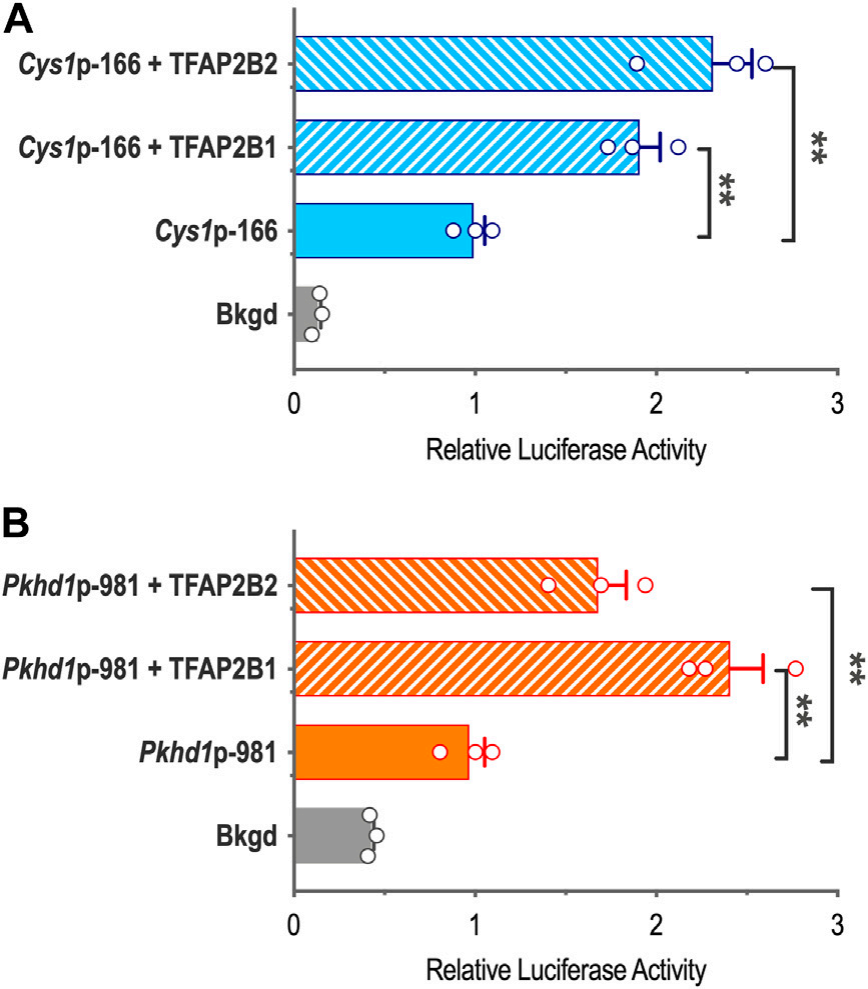
*Cys1* and *Pkhd1* promoter activation by TFAP2B1 and TFAP2B2. **(A)** The *Cys1*p-166 luciferase reporter construct (containing one TFAP2B binding site, see Figure 3) was transfected into mIMCD-3 cells with either TFAP2B2 or TFAP2B1 expression vectors. Relative luciferase activity (normalized to *Cys1*p-166 transfected alone) is shown. **(B)** A *Pkhd1*p-981 luciferase reporter construct (containing one TFAP2B binding site, similar to *Pkhd1*p-950 in Figure 4C) analyzed as described above. For both *Cys1* and *Pkhd1* constructs, TFAP2B1 and TFAP2B2 co-transfection significantly (**p < .01) increased luciferase activity compared to transfection of the *Cys1* or *Pkhd1* luciferase reporter alone. The error bar indicates S.E.M.

To characterize TFAP2B effects on *Cys1* and *Pkhd1* promoter reporter activity in the presence of overexpressed TFAP2B1 or TFAP2B2 isoforms, we cloned TFAP2B1 and TFAP2B2 coding sequences and generated expression vectors CMV-Tfap2b1 and CMV-Tfap2b2, which were co-transfected into mIMCD-3 cells together with either *Cys1*p-166 or *Pkhd1*p-981 luciferase reporter constructs. Both TFAP2B1 and TFAP2B2 increased *Cys1*p-166-linked luciferase activity, and TFAP2B2 was more effective than TFAP2B1 (Figure 4A). Similarly, both TFAP2B1 and TFAP2B2 enhanced luciferase activity from *Pkhd1*p-981. In this case, TFAP2B1 was the more active isoform (Figure 4B). We suggest that the observed differences in the magnitudes of activation of *Cys1* and *Pkhd1* promoters by TFAP2B1 and TFAP2B2 isoforms may reflect differences in the abundance of differentially active homo- and heterodimers that they may form. However, overexpression of either TFAP2B1 or TFAP2B2 in mIMCD-3 cells did not substantially impact the expression levels of endogenous *Pkhd1* and *Cys1* (Supplementary Figure S3).

**FIGURE 5:**
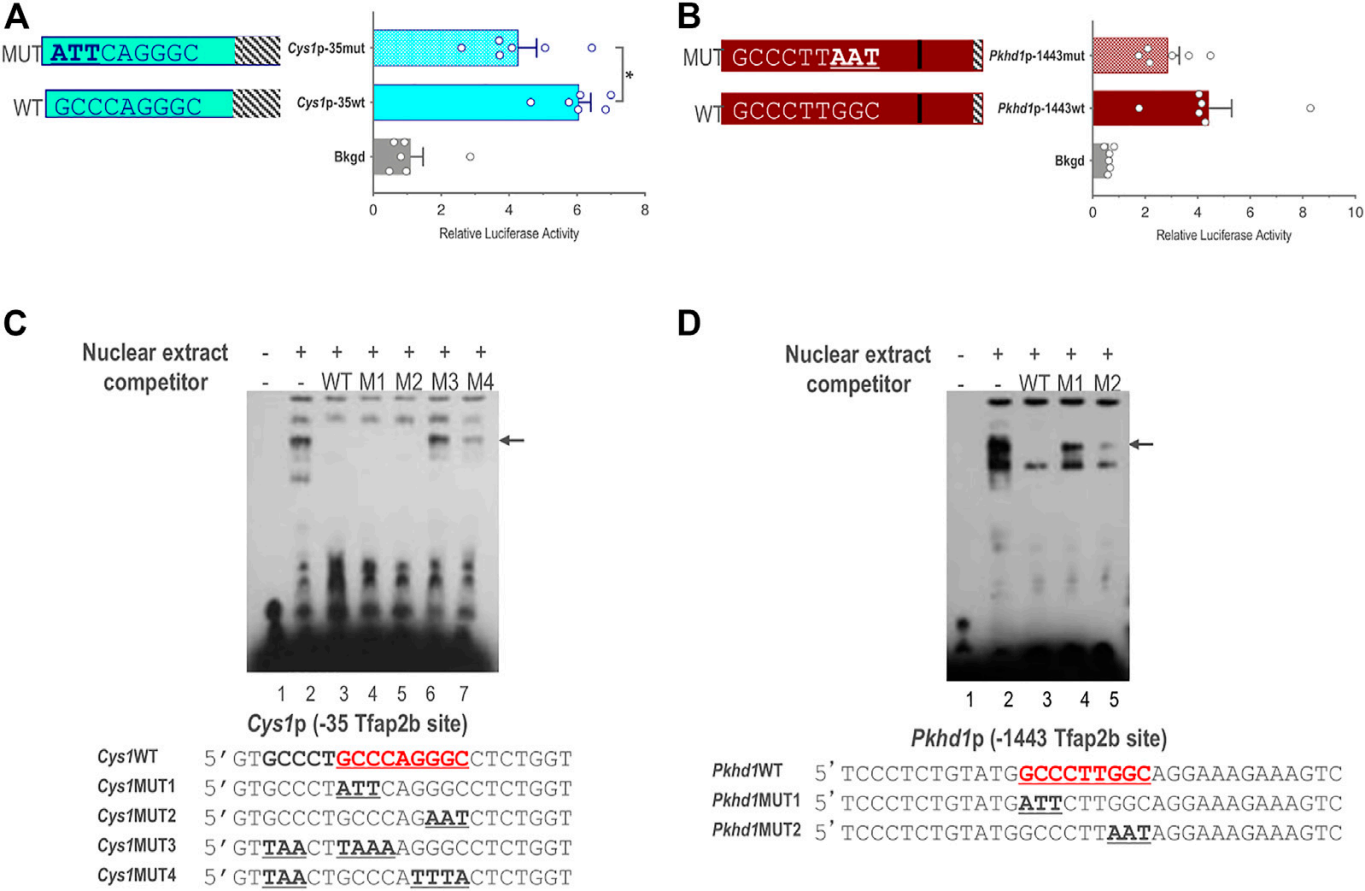
Endogenous TFAP2B binding to sites in *Cys1* and *Pkhd1* promoters. **(A)** Luciferase activity in mIMCD-3 cells transfected with *Cys1*p-35 reporter constructs bearing either WT or mutant versions of the proximal *Cys1* TFAP2B consensus binding site. **(B)** Luciferase assays in cells transfected with *Pkhd1*p-1443 reporter constructs with either WT or mutant forms of the distal *Pkhd1* TFAP2B binding site. **(C and D)** EMSA analysis comparing competitive binding of nuclear extract to WT vs. mutant forms of the *Cys1*p-35 **(C)** and *Pkhd1*p-1443 **(D)** TFAP2B site. WT TFAP2B binding site and mutated bp are shown in bold and underlined typeface. Arrows indicate shifted bands of interest. The error bar indicates S.E.M.; * indicates *p* < .05.

We further validated a role for TFAP2B in the regulation of *Cys1* and *Pkhd1* by assessing activity of *Cys1*p-35 and *Pkhd1*p-1443 luciferase reporter constructs in which the TFAP2B binding sites were mutated, such that conserved GCC nucleotides within the TFAP2B binding site were changed to ATT (Figures 5A, B). Mutation of the proximal TFAP2B binding site in *Cys1*p-35 resulted in lower luciferase activity (Figure 5A). Likewise, mutation of the distal TFAP2B binding site in the *Pkhd1*p-1443 *Pkhd1* upstream regulatory region showed a tendency to reduce luciferase activity, though this effect was not statistically significant (*p* = .14, n = 6) (Figure 5B). These findings support a role for TFAP2B in regulating *Cys1* and *Pkhd1* transcription.

### 3.6 Endogenous TFAP2B binds to the predicted *Cys1* and *Pkhd1* regulatory sequences

We used electrophoretic mobility shift assay (EMSA) to determine whether TFAP2B binds directly to the predicted sequences in the *Pkhd1* and *Cys1* promoters. mIMCD-3 cell nuclear extracts predicted to contain TFAP2B were incubated with biotin-labeled oligonucleotide probes with sequences either representing the proximal TFAP2B binding site from the *Cys1* (Figure 5C) or the *Pkhd1* promoter (Figure 5D). Binding of TFAP2B resulted in a mobility shift (retardation) of the oligonucleotide on the non-denaturing PAGE (Figure 5C, lane 2), indicated binding of endogenous TFAP2B to the oligonucleotides. Unlabeled oligonucleotides representing the wild-type (WT) TFAP2B binding sites (no avidin-based detection) showed competition with the labeled oligonucleotide for TFAP2B binding resulting in the elimination of the higher molecular weight band on the gel (Figure 5C, lane 3). These observations, together with the results from the reporter gene assays, confirm the presence of functional TFAP2B binding sites (Figure 5A). However, two oligonucleotides with GCC mutated to ATT/AAT on either side of the binding site also showed competition with the labeled WT oligonucleotide (Figure 5C, lanes 4-5).

We further examined the proximal TFAP2B binding region within the *Cys1* promoter by mutating another GCC motif five nucleotides upstream of the TFAP2B binding site (Figure 5C, oligo *Cys1*WT). Mutating this GCC to TAA, in tandem with mutation of the predicted TFAP2B binding site (Figure 5C, oligos *Cys1*MUT3 and *Cys1*MUT4), abrogated the ability of unlabeled mutant oligonucleotides to compete with labeled WT oligonucleotide probe (Figure 5C, lanes 6 and 7). These observations suggest that DNA sequences flanking the TFAP2B binding site may assist the attachment of TFAP2B to its binding site in the *Cys1* promoter. We note that the DNA sequences immediately upstream of the TFAP2B binding site in the *Cys1*WT, *Cys1*MUT1 and *Cys1*MUT2 oligos were derived from the mouse genome and differed from the vector-derived DNA sequences immediately upstream of the TFAP2B binding site in the *Cys1*p-35mut construct and these differences are responsible for the unanticipated binding of *Cys1*MUT1and *Cys1*MUT2.

In the case of *Pkhd1*, an oligonucleotide probe representing the distal upstream TFAP2B binding site was observed to undergo a mobility shift when incubated with mIMCD-3 cell nuclear extract (Figure 5D, lane 2), confirming TFAP2B binding to this site *in vitro.* Unlabeled WT oligonucleotide competed for TFAP2B binding (Figure 5D, lane 3) while two oligonucleotides with mutated TFAP2B binding sites did not (Figure 5D, lanes 4 and 5), confirming TFAP2B binding to the predicted sequences in the *Pkhd1* promoter. DNA sequences flanking the TFAP2B binding site in the *Pkhd1* promoter did not facilitate *in vitro* binding of TFAP2B to its binding site.

## 4 Discussion

While *PKHD1* mutations are the major cause of ARPKD in human, *Pkhd1* mutant mice do not recapitulate the ARPKD renal phenotype for yet undefined reasons. The *cpk* mouse, by contrast, is the best-characterized animal model which similarly resembles to that of human ARPKD. The renal cystic phenotype in *Tfap2b* ^*−/−*^ mice (Moser et al., 1997a; Moser et al., 2003; Wang et al., 2018; Lamontagne et al., 2022) and our results indicate that TFAP2B may activate both the *Pkhd1* and *Cys1* promoters. Identification of TFAP2B as a common activating transcription factor for *Cys1* and *Pkhd1* further support a functional link between these two cystogenes in collecting duct cells.

While patients with ARPKD due to *PKHD1* defects and *Pkhd1* mutant mice both have the liver ductal plate malformation phenotype, *TFAP2B/Tfap2b* apparently is not expressed in adult human and mouse livers or during embryonic development^1^ ^,^ ^2^ ^,^ ^3^. This could be either because *TFAP2B/Tfap2b* does not function in the liver or more likely, because cholangiocytes represent a small fraction (∼<5%) of the total liver epithelial cell mass. This issue remains unresolved as the scRNA-seq data in ENCODE database and published single-cell RNA-seq data (Aizarani et al., 2019) reveal absent or very low expression of *TFAP2A* and *TFAP2B* in adult human cholangiocytes; publicly available mouse scRNA-seq data (Tabula Muris database^4^) does not have an entry for cholangiocytes.

DNA binding sites of human TFAP2A, −B, −C, and −E transcription factors identified either through analysis of ChIP-seq data or SELEX experiments (Castro-Mondragon et al., 2022) are similar to TFAP2B binding sites we evaluated in the current report (Supplementary Figure S1). Furthermore, we have demonstrated similarity between DNA binding sites of human and mouse TFAP2B orthologues. Given the similarity in the DNA binding sites of the AP2 transcription factor family, the specificity in target gene activation appears to result from differential expression of TFAP2 family member(s) in specific cell types. For example, single cell RNA-seq experiments demonstrate *Tfap2b* expression in cortical collecting duct cells and *Tfap2a* expression in medullary collecting duct cells of adult mouse kidneys (Humphreys labor^5^) (Park et al., 2018; Ransick et al., 2019). Evaluation of conditional knockout mice clarify the roles of *Tfap2a* and *Tfap2b* in the developing and mature mouse kidney. Mice lacking functional *Tfap2a* in nephron progenitor cells have phenotypically normal kidneys while *Tfap2a* inactivation in adult medullary collecting ducts results in cystic dilatation (Lamontagne et al., 2022), suggesting that *Tfap2a* functions in the maintenance of medullary collecting duct cell terminal differentiation. In contrast, heterozygosity of *Tfap2b* in nephron progenitor cells causes progressive distal convoluted tubule abnormalities with associated renal fibrosis and cysts; whereas complete loss abolishes the development of distal convoluted tubules, and causes renal cysts, fibrosis, and early postnatal death (Marneros, 2020; Lamontagne et al., 2022). Therefore, *Tfap2a* and *Tfap2b* have non-redundant, distinct, spatiotemporal functions in discrete segments of the distal nephron.

We note that in both human *PKHD1*-related disease and the *Cys1* ^*cpk/cpk*^ mouse model of ARPKD, cystic disease is initiated in the fetal proximal tubule and transitions to collecting duct dilation as the predominant lesion in the perinatal period (Nakanishi et al., 2000). Based on these phenotypic observations and our data indicating that TFAP2B regulates *Pkhd1* and *Cys1,* we propose that *Tfap2b,* with *Pkhd1* and *Cys1,* are components of a transcriptional network that modulates renal tubular cell functional differentiation, which when disrupted leads to an ARPKD-like phenotype. However, there is complexity in the renal phenotype with differences between human and mice depending upon which gene is defective. Genetic defects in *TFAP2B/Tfap2b* genes result in ARPKD-like phenotype in mice (Moser et al., 1997a; Moser et al., 2003; Wang et al., 2018; Lamontagne et al., 2022) while humans with Char syndrome have no renal phenotype^6^. Genetic defects in *PKHD1/Pkhd1* genes result in ARPKD in humans, while mice have either no or mild renal phenotype (O’Connor and Guay-Woodford, 2016). Genetic defects in *CYS1/Cys1* genes result in ARPKD in both humans and mice (Yang et al., 2021). This phenotype variation suggests differential molecular functions of these genes in human and mouse renal epithelia. These differences reflect either susceptibility to cyst development in human kidneys or protective mechanisms in mouse kidneys and can be exploited for identifying therapeutic targets for ARPKD.

Our reporter gene assays are a simplified experimental system designed to test specific protein-DNA interactions. Expression of endogenous *Pkhd1* and *Cys1* in mIMCD-3 cells was not affected by overexpression of either TFAP2B1or TFAP2B2. Several factors could account for this result. First, mIMCD-3 cells originate from the inner medullary collecting duct cells, which do not express *Tfap2b* (Lamontagne et al., 2022) and may lack other transcription factors, such as KCTD1 (Marneros, 2020) and GAS41/YEATS4 (Ding et al., 2006) needed to activate expression of TFAP2B-regulated genes. Second, in addition to direct binding of upstream regulatory regions, TFAP2B could regulate *Pkhd1* and *Cys1* gene expression by modulating chromatin structure through interactions with YEATS4, which is a component of histone acetyltransferase machinery (Cai et al., 2003; Doyon et al., 2004).

Our *in vitro* findings set the stage for further studies to decipher the *in vivo* role for TFAP2B in the regulation of *Cys1* and *Pkhd1* expression. Such studies should include an analysis of the extent to which an overexpressed *Tfap2b*, *Pkhd1* or *Cys1* transgene can rescue the *Tfap2b* ^*−/−*^ cystic kidney phenotype, as we have recently demonstrated for the *cpk* mouse renal phenotype (Yang et al., 2021). Furthermore, from a clinical perspective, we speculate that among the approximately 20% of patients with ARPKD who lack mutations in *PKHD1*, *DZIP1* or *CYS1* coding sequences, there may be patients for whom disease is due to sequence variants in the TFAP2B binding sites of *PKHD1* or *CYS1*. Alternatively, these patients may carry novel mutations directly affecting *TFAP2B*-regulated genes that are expressed in renal collecting duct epithelia.

Characterization of TFAP2B regulation of *Pkhd1*, *Cys1* and potentially other cystogenic genes will expand our understanding of transcriptional networks that modulate renal tubular cell functional differentiation and provide new mechanistic insights into how disruption of these regulatory networks contributes to the ductal dilatation that is the hallmark of recessive PKD.

## Data Availability

The original contributions presented in the study are included in the article/Supplementary Material, further inquiries can be directed to the corresponding author.

## Footnotes

1
https://www.gtexportal.org/home/gene/TFAP2B

2
https://www.ncbi.nlm.nih.gov/gene/702

3
https://www.ncbi.nlm.nih.gov/gene/21419

4
https://tabula-muris.ds.czbiohub.org

5
http://humphreyslab.com/SingleCell/search.php

6
https://monarchinitiative.org/gene/HGNC:11743#phenotype
