# Supplementary material for: Transcription factor Ap2b regulates the mouse autosomal recessive polycystic kidney disease genes, Pkhd1 and Cys1
Source: Front Mol Biosci. 2023 Jan 12;9:946344. doi: 10.3389/fmolb.2022.946344 (PMC9877354; doi:10.3389/fmolb.2022.946344)
Supplement: Supplementary file 1 [file DataSheet1.pdf]

Supplementary Material

1 Supplementary Data

1.1 Supplementary Figures

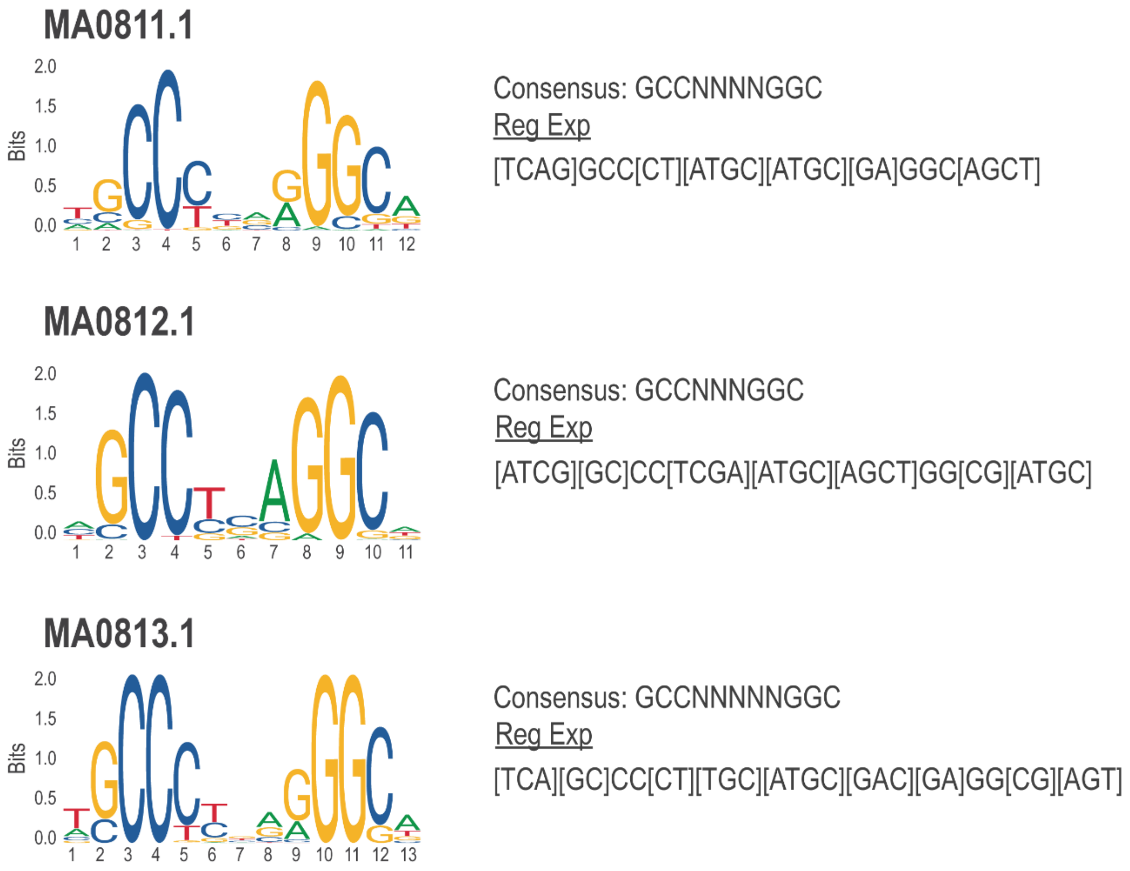

Supplementary Figure S1. TFAP2B binding sites.

LOGO representations of TFAP2B position similarity matrices from the JASPAR database, TFAP2B consensus binding sites and corresponding regular expressions.

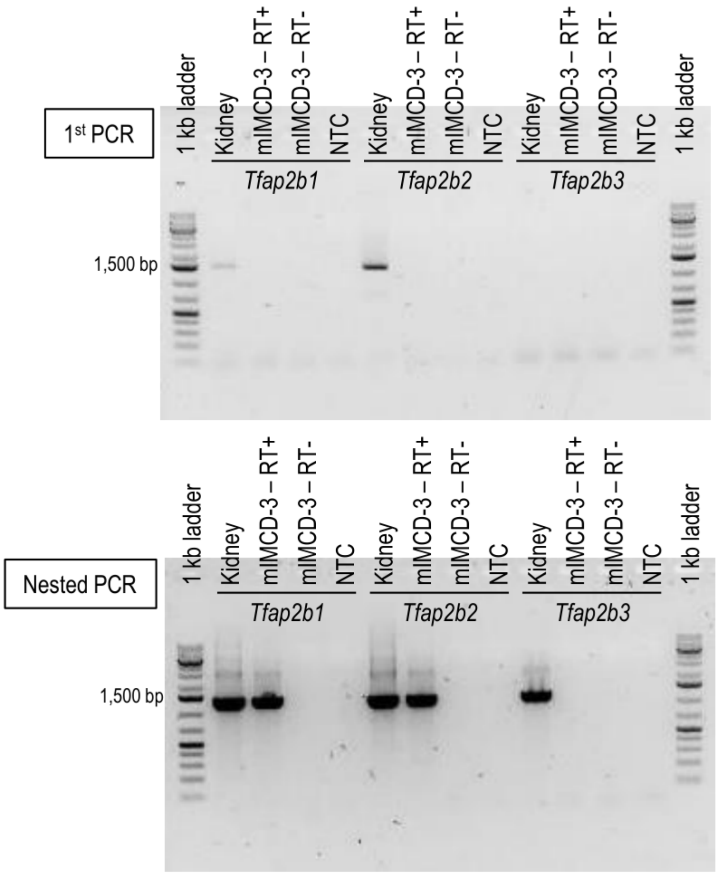

Supplementary Figure S2. RT-PCR analysis of *Tfap2b* mRNA expression in mouse kidney tissue and mIMCD-3 cells.

(Upper panel) 1st (*i.e.* initial) PCR amplification of cDNA samples generated from mouse kidney tissue and mIMCD-3 cell total RNA, using primers specific for *Tfap2b1*, *Tfap2b2*, and *Tfap2b3* mRNA isoforms (see Supplementary Tables S1 and S2 for primers and PCR reaction conditions, respectively). RT+ and RT- indicate reactions performed with and without reverse transcriptase, respectively. NTC, no template control. *Tfap2b1* and *Tfap2b2* mRNAs were detected in kidney tissue, but not in mIMCD-3 cells. *Tfap2b3* mRNA was not detected in either kidney or cell line.

(Lower panel) Nested PCR performed with 1st PCR (see Supplementary Tables S1 and S2 for primers and PCR reaction conditions, respectively). Nested PCR detected *Tfap2b1* and *Tfap2b2* expression in kidney and mIMCD-3 cells, and *Tfap2b3* expression in kidney but not in cells.

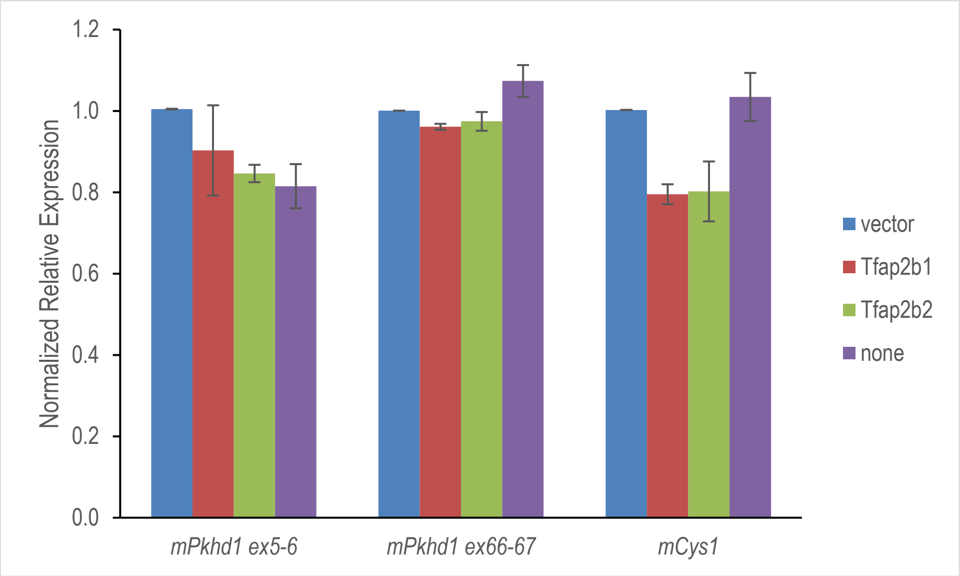

Supplementary Figure S3. Expression of *Pkhd1* and *Cys1* in mIMCD-3 cells overexpressing either TFAP2B1 or TFAP2B2.

Quantitative RT-PCR was used to determine relative mRNA expression levels of *Pkhd1* and *Cys1* in the mIMCD-3 cells overexpressing either TFAP2B1 or TFAP2B2.

1.2 Supplementary Tables

Supplementary Table S1. Primers used for amplification of *Tfap2b* isoforms from cDNA prepared from mIMCD-3 cells and mouse kidney tissue.

	Name	Sequence
1st PCR	Tfap2b1/3_F1	TCTGCTCCTCACATGAATGC
	Tfap2b2_F1	ATTTTGAGCAGTAACCAGGC
	Tfap2b1/2_R2	TTTCTCCTCCTTGTCGCCAG
	Tfap2b1/2/3_R1	AGTAAGAGCAAGGCGCATCC
Nested	Tfap2b1/3_F2	AGGCTGCGATCATGCTCTG
	Tfap2b2_F2	CCAGATGTTAGTCCACACCT
	Tfap2b1/2_R1	CTTGTCGCCAGTTTTACTAC
	Tfap2b3_R1	CGTTGCCCTGGGCCTTC

Supplementary Table S2. PCR conditions used for amplification of *Tfap2b* isoforms from cDNA prepared from mIMCD-3 cells and mouse kidney tissue.

	Temperature	Time	Cycles (1st PCR)	Cycles (Nested PCR)
Initial denaturation	95°C	2 min	1	1
Denaturation	95°C	30 sec	35	30
Annealing	55°C	30 sec		
Elongation	72°C	2 min 30 sec		
Final elongation	72°C	5 min	1	1
Cooling	12°C	Unlimited time		

Supplementary Table S3. Primers used for amplification of *Cys1* upstream regulatory region.

Plasmid Name	Forward	Reverse
*Cys1*p-2858	CTAGCTAGCATGATGCTGGGGATGGAACCAGGC	CCCAAGCTTGGAGACTAGCACTGTCGGAAAGGAGG
*Cys1*p-1091	CTAGCTAGCTCACCTGCCTATGCTCCACGAGTG	CCCAAGCTTGGAGACTAGCACTGTCGGAAAGGAGG
*Cys1*p-524	CTAGCTAGCCATTGGGTTGGCCAGTACGGATGC	CCCAAGCTTGGAGACTAGCACTGTCGGAAAGGAGG
*Cys1*p-166	CTAGCTAGCTCGCAATCTTCCACAATCTAACCAC	CCCAAGCTTGGAGACTAGCACTGTCGGAAAGGAGG
*Cys1*p-35	CTAGCTAGCGCCCAGGGCCTCTGGTACACGCCT	CCCAAGCTTGGAGACTAGCACTGTCGGAAAGGAGG
*Cys1*p-26	CTAGCTAGCCTCTGGTACACGCCTCTCAACCTC	CCCAAGCTTGGAGACTAGCACTGTCGGAAAGGAGG

The *Cys1* TSS genomic coordinate is Chr12:24,681,806 from the GRCm38/mm10 mouse genome assembly.

Supplementary Table S4. PCR conditions used for amplification of *Cys1* and *Pkhd1* regulatory regions.

	Temperature	Time	Cycles
Initial denaturation	94°C	2 min.	1
Denaturation	94°C	10 sec.	10
Annealing	65°C	30 sec.	
Elongation	68°C	4 min.	
Denaturation	94°C	15 sec.	25
Annealing	67°C	30 sec.	
Elongation	68°C	4 min. + 20 sec. at each successive cycle	
Final Elongation	68°C	7 min.	1
Cooling	4°C	Unlimited time	

Supplementary Table S5. Primers used for amplification of *Pkhd1* upstream regulatory region.

Plasmid Name	Forward	Reverse
*Pkhd1*p-1443	GGGGTACCGTATGGCCCTTGGCAGGAAAGAAAG	GAAGATCTACCTAACTCGCTTGTGTCCTGGC
*Pkhd1*p-1435	GGGGTACCGAAAGTCTGTTAATGAAAGTCTCAC	GAAGATCTACCTAACTCGCTTGTGTCCTGGC
*Pkhd1*p-981	GAAGATCTAAGGAAAGGGGAAAAGCCCAAACC	CCCAAGCTTACCTAACTCGCTTGTGTCCTGGC
*Pkhd1*p-950	GAAGATCTGCCCCAGGCATTCTCAGTTTCAA	CCCAAGCTTACCTAACTCGCTTGTGTCCTGGC
*Pkhd1*p-941	GAAGATCTATTCTCAGTTTCAATACTTTG	CCCAAGCTTACCTAACTCGCTTGTGTCCTGGC

2 Supplementary Materials and Methods

mIMCD-3 cells were transfected with pCMV-Myc-N (vector), CMV-Tfap2b1 (Tfap2b1), or CMV-Tfap2b2 (Tfap2b2) by Lipofectamine 2000 Transfection Reagent. After 48 hr. transfection, total RNA were isolated with RNeasy Mini Kit and synthesized 1st strand cDNA with SuperScript III First-Strand Synthesis SuperMix as described in Materials and Methods. Quantitative RT-PCR were performed on a QuantStudio 7 Flex Real-Time PCR System (Thermo Fisher Scientific) using the default program. The PCR was performed on cDNA templates using Power SYBR Green PCR Master Mix (Thermo Fisher Scientific, Cat. No. 4368706) and primers specific for sequences in *Pkhd1* exons 5-6 (forward: 5'- GTC TCT TCC ATC AGA AGC AGA TGC -3'; reverse: 5'- GGG TAA ACT TGA TAT AAA ACA GG -3'), Pkhd1 exons 66-67 (forward: 5'-CCA GAA GAC ATA

TCT GAA TCC CAG GC-3'; reverse: 5'-AGC AAG AGA TCC TGG AAC ACA GGT-3'), and *Cys1* (forward: 5'- GTC CAT GAA TCC TCA GAA CAC AAC C -3'; reverse: 5'- CTC AGC CAT TCG GTA GAC ACT C -3'). *Peptidylprolyl isomerase A* (*PPIA*) was used for normalization (forward: 5'- AGC ACT GGA GAG AAA GGA TT -3'; reverse: 5'- ATT ATG GCG TGT AAA GTC ACC A -3') (Arensdorf and Rutkowski, 2013). Results were analyzed using QuantStudio Real-Time PCR Software and the ΔΔCt method (Livak and Schmittgen, 2001). Experiments were repeated twice independently. The error bars indicate S.E.M.

3 References

Arensdorf, A.M., and Rutkowski, D.T. (2013). Endoplasmic reticulum stress impairs IL-4/IL-13 signaling through C/EBPβ-mediated transcriptional suppression. *J Cell Sci* 126(Pt 17), 4026-4036. doi: 10.1242/jcs.130757.

Livak, K.J., and Schmittgen, T.D. (2001). Analysis of relative gene expression data using real-time quantitative PCR and the 2(-Delta Delta C(T)) Method. *Methods* 25(4), 402-408. doi: 10.1006/meth.2001.1262.
